# Differences in and Correlates of Sexual Function in Infertile Women with and without Polycystic Ovary Syndrome

**DOI:** 10.22074/IJFS.2021.6206

**Published:** 2021-01-27

**Authors:** Azadeh Akbari Sene, Bita Tahmasbi, Farideh Keypour, Hadi Zamanian, Fahimeh Golbabaei, Mohammadali Amini-Tehrani

**Affiliations:** 1 Shahid Akbarabadi Clinical Research Development Unit (ShACRDU), Iran University of Medical Sciences (IUMS), Tehran, Iran; 2 School of Health, Qom University of Medical Sciences, Qom, Iran; 3 Health Psychology and Behavior Medicine Research Group, Student Scientific Research Center, Tehran University of Medical Sciences, Tehran, Iran; 4 School of Nursing and Midwifery, Iran University of Medical Sciences (IUMS), Tehran, Iran; 5 Department of Psychology and Education, University of Tehran, Tehran, Iran

**Keywords:** Infertility, Polycystic Ovary Syndrome, Sexual Dysfunction, Women

## Abstract

**Background:**

The aim of this study was to examine sexual function and its correlates among infertile women with polycystic ovary syndrome (PCOS) in comparison with their non-PCOS counterparts.

**Materials and Methods:**

In this case-control study, 209 infertile women (116 PCOS and 93 non-PCOS) from Tehran, Iran, were evaluated in February and March 2018. Female sexual function index (FSFI), hormonal status, and docu- mented reports of hyperandrogenic manifestations of the patients were investigated.

**Results:**

The mean age of the patients was 32.00 ± 5.00 years old. Eighty-four (40.2%) patients including 42.2% of the PCOS patients and 37.6% of the non-PCOS cases (P>0.05), were suspected of female sexual dysfunction (FSD). The most impaired functions in both groups were desire and arousal. Sexual function was not significantly different between the groups. However, PCOS women had more orgasm problems and acne worsened their sexual function. To- tal FSFI was positively associated with prolactin level but negatively associated with central obesity in the non-PCOS group; it was negatively correlated with marital duration in the PCOS group. Luteinizing hormone (LH) and pain, pro- lactin level and lubrication, and central obesity and arousal were correlated in the non-PCOS women. Prolactin level and orgasm, marital duration and arousal, and marital duration and the total FSFI were correlated in the PCOS women.

**Conclusion:**

Sexual function was similarly low in infertile PCOS and non-PCOS women. However, orgasm problems and the negative effect of acne varied between the two groups. Further investigations may target how hormonal profile may affect sexual function. Practitioners should scrutinize the specific impaired sexual domains and their correlated conditions in PCOS women, notably orgasm, acne, and prolactin level. Interventions should be well tailored based on particular needs of infertile PCOS women.

## Introduction

Infertility is a disease of the reproductive system that is defined as “the failure to achieve a clinical pregnancy after 12 months or more of regular unprotected sexual intercourse” (1). In the Iranian population, recent estimations indicated that lifetime primary infertility rates, based on clinical, epidemiological, and demographic definitions set by the World Health Organization, are respectively 20.2, 12.8, and 9.2%, while that of secondary infertility is 4.9% (2). One of the common disorders linked to infertility and associated with ovulation problems is polycystic ovary syndrome (PCOS). The definition of PCOS by the United States National Institutes of Health entails anovulation and hyperandrogenemia. However, the European Society of Human Reproduction and Embryology/American Society for Reproductive Medicine (ESHRE/ASRM) defines PCOS as polycystic ovaries diagnosed based on ultrasound (3). Studies have reported that up to 83% of infertile Iranian women have PCOS, and an infertility rate of 8-73% in PCOS patients was shown (4).

Sexual life of infertile women is a pivotal research area because infertility is associated with an increased risk of female sexual dysfunction (FSD) (5). Sexual function involves various domains, including sexual desire, arousal, vaginal lubrication, experiencing orgasm, coital satisfaction, and feeling of pain during intercourse (6). Infertile couples are more prone to develop sexual dysfunction. A recent estimation in infertile Iranian women, for instance, found that 64.3% of the patients developed sexual dysfunction, with sexual desire and vaginismus having the highest and lowest rates, respectively (7). Another study suggested that the sexual behavior of infertile Iranian women is severely narrowed to produce pregnancy (8) and that infertility causes Iranian women to suffer from serious problems in various domains, including sexuality (9). More to the point, sexual dysfunction may considerably affect mental health as well as the sexual quality of life (10). Furthermore, women’s sexual problems may even escalate if they concurrently suffer from PCOS (11). That is, the sexuality of women with PCOS may become even more compromised, especially as a result of obesity, hirsutism, baldness, and acne (12). Moreover, sexual problems are suggested as the possible causes of discrepancies in perceptions of infertile women with and without PCOS towards their sexual life and function (13).

Although various studies have addressed the sexual problems experienced by women with PCOS (14, 15), few studies have focused on sexual issues and their correlates when such patients simultaneously suffer from infertility. Recent meta-analyses have determined similar sexual function between PCOS women and healthy controls (16, 17). However, it can be argued that further studies investigating possible effects of comorbid PCOS and infertility are warranted, especially in the Iranian population. Moreover, investigating possible differences in sexual problems and their correlates between infertile PCOS patients and their non-PCOS counterparts may provide more knowledge about the specific role that comorbid PCOS and infertility play in Iranian women life.

The present study, therefore, investigated the differences between infertile Iranian women with and without PCOS in terms of sexual function. It also aimed to evaluate the degree to which hormonal, anthropomorphic, and hyperandrogenic manifestations may be correlated with the sexual function of these groups of women.

## Materials and Methods

### Study design and sampling

This case-control study involved infertile PCOS women as the case group and their infertile non-PCOS counterparts as the control group using a convenience sampling method. Patients visiting two infertility centers in Tehran, Iran were recruited. The study was introduced and explained to the patients to obtain their informed consent. The questionnaires were administered using the interviewer-administered method. Overall, 216 infertile women were recruited in February and March of 2018.

The sample size was determined for two independent samples using G*Power software V.3 (18). The estimation setting was set to a medium effect size (0.50), a significance level (α) of 0.05 (two-tailed), a power of 0.80, and an allocation ratio of 1. The calculation suggested that a sample size of 128 participants (64 for each group) could achieve the actual power of 0.803. The inclusion criteria were as follows: age above 18 years and diagnosis of infertility for both groups, and diagnosis of PCOS for the PCOS group. PCOS was diagnosed based on the international evidence-based guideline for the assessment and management of PCOS, 2018 (19). This guideline identifies the condition in adult women if two of the three conditions of androgen excess, ovulatory dysfunction, and polycystic ovarian morphology are present. Ultrasound is required if either androgen excess or ovulatory dysfunction is not present. Certain disorders, including thyroid disease (based on thyroid-stimulating hormone (TSH) level), hyperprolactinemia (based on prolactin level), and non-classic congenital adrenal hyperplasia (based on 17-hydroxyprogesterone (17-OHP) level), were ruled out by clinical judgment.

The following exclusion criteria were considered: psychiatric disorders; severe emotional problems in the past six months; consumption of oral contraceptive pills, gonadotropin-releasing hormone (GnRH) agonists, or insulin sensitizers in the past six months; chronic cardiovascular diseases; primary or secondary vaginismus and dyspareunia; pelvic mass; active genital infection; external vaginal anomalies; pelvic endometriosis; and partner’s sexual dysfunction.

### Measures

A checklist was devised to survey the participants’ demographic information, including the patient’s age, occupation, and education, the spouse’s age and education, and the duration of their marriage. Clinical information including duration of infertility, duration of treatment, central obesity (waist-to-hip ratio), body mass index (BMI), and hyperandrogenic manifestations, including the presence of acne, hirsutism (Ferriman-Gallwey score), and baldness (i.e. male-pattern hair loss), was also collected. 

On the third day of the menstrual cycle (induced by 100-200 mg progesterone in oil injection in amenorrheic patients), a baseline vaginal ultrasound examination was performed, and serum follicle-stimulating hormone (FSH), luteinizing hormone (LH), TSH, and prolactin levels were measured using immunoradiometric assays (Izotop, Budapest, Hungary). Dehydroepiandrosterone sulfate (DHEAS), 17-OHP, and total testosterone (TT) were measured using enzyme immunoassays (Diagnostics Biochem Canada Inc., London, Canada). 

The Female Sexual Function Index (FSFI) (20) was employed to assess the patients’ sexual function. This 19-item questionnaire assesses six domains of sexual desire (two items, domain factor 0.6), arousal (four items, domain factor 0.3), lubrication (four items, domain factor 0.3), orgasm (three items, domain factor 0.4), satisfaction (four items, domain factor 0.4), and pain (three items, domain factor 0.4) for a comprehensive evaluation of the female sexual response cycle. Each domain’s raw score is multiplied by its domain factor, yielding a possible score in the range of 0 to 6, except for desire, which has a range of 1.2 to 6. According to the score which ranges 1.2-36, higher total scores indicate better sexual function. The questionnaire can identify women who had no sexual encounters during the preceding four weeks. The Persian version of the FSFI was approved as a valid and reliable screening and assessment instrument, indicating a Cronbach’s alpha of 0.93 and test-retest reliability of 0.83 (21). The instrument showed a Cronbach’s alpha of 0.89 in the current dataset. The second and fifth authors administered the questionnaires using the interviewer-administered method by asking patients to choose the response that best described their status.

FSD was identified based on FSFI scores. In an original study by Rosen, scores lower than 26.55 indicated a diagnosis of FSD with a specificity of 0.73 and a sensitivity of 0.89 (20). In addition, a raw score below 3.9 indicated sexual dysfunction in each domain (22).

### Ethical considerations

This study was performed according to the Declaration of Helsinki, and the Ethics Committee of Iran University of Medical Sciences approved the study protocol (ID: IR.IUMS.FMD.REC.1396.9311290023). Informed consent was obtained from all participants.

### Statistical analysis

Statistical analysis was performed using IBM SPSS Statistics for Windows, Version 24.0 (IBM Corp., Armonk, NY, USA) (23). Missing data were handled using the pairwise deletion method. Since the scores showed a nonnormal distribution, non-parametric statistics was adopted. The Mann-Whitney U test and the Chi-square test for comparing the groups, as well as logistic regression for predicting FSD via hyperandrogenic manifestations, were conducted. Spearman’s Rho was also calculated to determine the correlation of patient’s age, duration of infertility, duration of treatment, BMI, central obesity, levels of FSH, TSH, LH, prolactin, TT, DHEAS, and 17-OHP (all in mcg/L), and LH/FSH ratio with the total FSFI score and the domains. Two-tailed P value was set at <0.05.

## Results

### Sample characteristics

Among the 216 patients recruited in this study, seven (0.03%) patients were sexually inactive based on the FSFI and were excluded. Therefore, 209 infertile women, including 116 PCOS patients and 93 non-PCOS patients, remained in the study

Table 1 presents the demographic and clinical characteristics of the patients. The mean age was 32.00 ± 5.00 years, with the PCOS group being older (P<0.001). Central obesity was more frequent in the PCOS group (P<0.01), and there was a higher degree of anovulation as the cause of infertility (P<0.001) in this group. Conversely, infertility in the non-PCOS group was more often found to be unexplained or due to tubal causes (P<0.01).

### Evaluation of female sexual function

Table 2 presents the sexuality domains for both groups. Arousal had the lowest score in the PCOS group (3.69 ± 1.23) and non-PCOS group (3.67 ± 1.32). On the other hand, the highest mean score was related to satisfaction in the PCOS group (5.06 ± 1.00) and non-PCOS group (5.11 ± 0.95). The total FSFI for in our sample population was 27.15 ± 4.30, with 26.97 ± 4.73 in the PCOS group and 27.38 ± 3.72 in the non-PCOS group. FSD was diagnosed in 40.2% of the participants, including 42.2% of the PCOS patients and 37.6% of the non-PCOS patients.

### Sexual function comparison

Table 3 presents comparisons between the PCOS and nonPCOS groups in terms of sexual function. Based on the raw scores for sexual function, there were no significant differences between the two groups (P=0.325 to 0.975). Also, the two groups had no significant difference in terms of FSD (P=0.500). Based on the categorization of sexual dysfunction in the domains (score<3.9), the two groups only showed a significant difference in orgasm problems (P=0.035).

### Hyperandrogenic manifestations and sexual function

According to Table 3, acne (51.7%), baldness (41.4%), and hirsutism (57.8%) were more commonly found in the infertile PCOS patients (P<0.001). Acne was the sole impactful hyperandrogenic manifestation, increasing the odds of FSD by 1.87 [1.02, 3.43] (P=0.042) in the total sample and 2.18 [1.01, 4.68] (P=0.046) in the PCOS group.

### Hormone comparison

Table 4 presents the results of the hormone tests in both groups. Group differences were seen in FSH, which was higher in the non-PCOS group, and LH, which was higher in the PCOS group (P<0.001). The LH/FSH ratio was higher in the PCOS group to a statistically significant degree (P<0.001).

### Sexual function correlates

In the non-PCOS patients, there were significant relationships between LH and pain (n=87, rho=0.26, P=0.015), prolactin level and lubrication (n=85, rho=0.22, P=0.048), prolactin level and total FSFI (n=85, rho=0.22, P=0.045), and central obesity and arousal (n=93, rho=-0.26, P=0.013). In the PCOS group, marital duration and arousal (n=103, rho=-0.31, P=0.001), marital duration and total FSFI (n=103, rho=-0.25, P=0.013), and prolactin level and orgasm (n=114, rho=-0.23, P=0.012) were correlated.

### Confounding effect of age

Because the mean ages of the two groups were statistically significantly different (P<0.001, Table 1), the analyses were repeated using linear regression analysis, including the PCOS group as the dummy variable and age as the covariate. There were no significant changes in the pattern of the comparisons. Additionally, controlling for age, partial correlations were estimated between each pair of hormones and sexuality domains. Consequently, the results indicated only negligible changes to the zero-order correlations. Therefore, the previously reported differences and correlations were shown to be valid.

**Table 1:** Demographic and clinical information of the study sample


Variables	Totaln=209	PCOSn=116	Non-PCOS n=93	Test statistic	P value

Patient’s age (Y)	32.00 ± 5.00	31.00 ± 5.00	34.00 ± 6.00	z=-4.06	<0.001^a^
Spouse’s age (Y)	36.00 ± 6.00	35.00 ± 6.00	37.00 ± 6.00	z=-3.57	<0.001^a^
Duration of marriage (Y)	6.70 ± 4.40	6.40 ± 4.20	7.10 ± 4.70	z=0.96	0.339^a^
Duration of infertility (Y)	4.20 ± 3.40	4.00 ± 3.50	4.40 ± 3.40	z=-1.50	0.133^a^
Duration of treatment (Y)	0.80 ± 0.61	0.79 ± 0.64	0.82 ± 0.58	z=0.98	0.329^a^
BMI	26.63 ± 4.10	26.66 ± 3.85	26.58 ± 4.43	z=0.76	0.448^a^
Central obesity	0.90 ± 0.06	0.91 ± 0.06	0.89 ± 0.06	z=2.61	0.009^a^
Patients’ education					
Illiterate	4 (1.9)	2 (1.7)	2 (2.2)	χ^2^[5] = 8.33	0.139^b^
Primary	13 (6.2)	6 (5.2)	7 (7.5)		
Secondary	29 (13.9)	20 (17.2)	9 (9.7)		
Diploma	74 (35.4)	40 (34.5)	34 (36.6)		
University	53 (25.4)	34 (29.3)	19 (20.4)		
Missing data	36 (17.2)	14 (12.1)	22 (23.7)		
Patients’ job					
Housewife	149 (71.3)	85 (73.3)	64 (68.8)	χ^2^[3] =5.00	0.172^b^
Home-based	2 (1.0)	2 (1.7)	0 (0.0)		
Employed	26 (12.4)	16 (13.8)	10 (10.8)		
Missing data	32 (15.3)	13 (11.2)	19 (20.4)		
Spouses’ education					
Illiterate	4 (1.9)	3 (2.6)	1 (1.1)	χ^2^[5] = 9.12	0.104^b^
Primary	14 (6.7)	7 (6.0)	7 (7.5)		
Secondary	45 (21.5)	27 (23.3)	18 (19.4)		
Diploma	65 (31.1)	36 (31.0)	29 (31.2)		
University	41 (19.6)	28 (24.1)	13 (14.0)		
Missing data	40 (19.1)	15 (12.9)	25 (26.9)		
Infertility type					
Primary	125 (59.8)	75 (64.7)	50 (53.9)	χ^2^[2] = 2.95	0.229^b^
Secondary	63 (30.1)	31 (26.7)	31 (33.3)		
Missing data	21 (10.0)	9 (7.8)	12 (12.9)		
Previous treatment					
None	51 (24.4)	33 (28.4)	18 (19.4)	χ^2^[1] = 3.57	0.168^b^
Induce	65 (40.4)	44 (46.8)	21 (31.3)	χ^2^[1] = 3.89	0.049^b^
IUI	65 (40.4)	32 (34.0)	33 (49.3)	χ^2^[1] = 3.76	0.052^b^
IVF/ICSI	29 (18.0)	15 (16.0)	14 (20.9)	χ^2^[1] = 0.65	0.442^b^
Missing data	48 (23.0)	22 (19.0)	26 (28.0)		
Infertility cause^c^					
Anovulation	128 (61.2)	110 (94.8)	18 (19.4)	χ^2^[1] = 123.87	<0.001^b^
Tubal	26 (12.4)	6 (6.0)	19 (20.4)	χ^2^[1] = 9.82	0.002^b^
Unexplained	17 (8.1)	3 (2.6)	14 (15.1)	χ^2^[1] = 10.73	0.001^b^
Missing data	42 (20.1)	0 (0.0)	42 (45.2)		


Data are presented as mean ± SD or n (%). IUI; Intrauterine insemination, IVF; *In vitro* fertilization, ICSI; Intrauterine insemination, PCOS: Polycystic ovary syndrome, BMI; Body mass index, ^a^ ; Mann-Whitney U (Asymp. P), ^b^; Pearson’s Chi-square test (2-sided), and^ c^ ; Anovulation and tubal categories are not mutually exclusive. The bolded P indicates a significant difference.

**Table 2:** Comparisons of sexual function


Variables	Total n=209			PCOS n=116			Non-PCOS n=93			z statistic	P^a^	χ^2^[1]	P^b^
	M ± SD	Min, Max	FSD n (%)	M ± SD	Min, Max	FSDn (%)	M ± SD	Min, Max	FSD n (%)				

Desire	3.79 ± 1.05	1.20, 6.00	117 (56.0)	3.78 ± 1.01	1.20, 6.00	67 (57.8)	3.81 ± 1.09	1.20, 6.00	50 (53.8)	-0.24	0.792	0.33	0.563
Arousal	3.68 ± 1.27	1.20, 6.00	91 (43.5)	3.69 ± 1.23	1.20, 6.00	56 (48.3)	3.67 ± 1.32	1.20, 6.00	35 (37.6)	-0.29	0.775	2.38	0.123
Lubrication	4.99 ± 1.12	1.20, 6.00	34 (16.3)	4.92 ± 1.15	1.20, 6.00	21 (18.1)	5.07 ± 1.07	1.20, 6.00	13 (14.0)	-0.99	0.325	0.65	0.442
Orgasm	4.60 ± 1.08	1.20, 6.00	48 (23.0)	4.52 ± 1.17	1.20, 6.00	33 (28.4)	4.68 ± 0.95	1.20, 6.00	15 (16.1)	-0.61	0.539	4.43	0.035
Satisfaction	5.08 ± 0.98	2.40, 6.00	26 (12.4)	5.06 ± 1.00	2.40, 6.00	15 (12.9)	5.11 ± 0.95	2.40, 6.00	11 (11.8)	-0.34	0.738	0.06	0.810
Pain	5.01 ± 1.05	1.60, 6.00	34 (16.3)	5.00 ± 1.09	1.60, 6.00	22 (19.0)	5.04 ± 1.00	1.60, 6.00	12 (12.9)	-0.03	0.978	1.39	0.238
FSFI	27.15± 4.30	15.90, 36.00	84 (40.2)	26.97 ± 4.73	15.90, 36.00	49 (42.2)	27.38 ± 3.72	16.60, 34.50	35 (37.6)	-0.29	0.774	0.46	0.500


PCOS; Polycystic ovary syndrome, M; Mean, SD; Standard deviation, Min; Minimum, Max; Maximum, FSFI; Female sexual function index, FSD; Female sexual dysfunction (FSFI below 26.55 for global FSD and below 3.9 for domain FSD), ^a^ ; Mann-Whitney U (Asymp. P) on the raw scores, and ^b^; Pearson’s Chi-square test (2-sided) on the FSD categorizations. The bolded P indicates a significant difference.

**Table 3:** Predicting FSD based on hyperandrogenic manifestations


Variables		Total n=209	PCOS n=116	Non-PCOS n=93	Total		PCOS	
					Unadjusted^a^	Adjusted^b^	Unadjusted^a^	Adjusted^b^
			n (%)			Odds [95%CI]		

Acne								
	None	135 (64.6)	54 (46.6)	81 (87.1)	1.87 [1.02, 3.43]	2.15 [1.12, 4.11]	2.18 [1.01, 4.68]	2.22 [1.00, 4.89]
	Mild	48 (23.0)	44 (37.9)	4 (4.3)				
	Moderate	13 (6.2)	13 (11.2)	0 (0.0)				
	Severe	3 (1.4)	3 (2.6)	0 (0.0)				
Baldness								
	None	151 (72.2)	66 (56.9)	85 (91.4)	0.89 [0.45, 1.73]	0.77 [0.38, 1.56]	0.77 [0.36, 1.64]	0.62 [0.28, 1.27]
	Mild	28 (13.4)	28 (24.1)	0 (0.0)				
	Moderate	14 (6.7)	14 (12.1)	0 (0.0)				
	Severe	6 (2.9)	6 (5.2)	0 (0.0)				
Hirsutism								
	None	129 (61.7)	47 (40.5)	82 (88.2)	1.34 [0.74, 2.42]	1.44 [0.77, 2.71]	1.23 [0.57, 2.63]	1.22 [0.56, 2.67]
	Mild	44 (21.1)	41 (35.3)	3 (3.2)				
	Moderate	18 (8.6)	18 (15.5)	0 (0.0)				
	Severe	8 (3.8)	8 (6.9)	0 (0.0)				


FSFI; Female sexual function index, FSD; Female sexual dysfunction (FSFI below 26.55), PCOS; Polycystic ovary syndrome, CI; Confidence interval,^ a^ ; The unadjusted models included each hyperandrogenic manifestation as an independent variable and FSD as a dependent variable, and ^b^; The models were adjusted for age. Bolded results indicate significant (P<0.05)

**Table 4:** Comparisons of laboratory results


Hormones^a^	Total n=209			PCOS n=116			Non-PCOS n=93			z statistic	P^b^
	n	M ± SD	Min, Max	n	M ± SD	Min, Max	n	M ± SD	Min, Max		

FSH	201	4.93 ± 3.27	0.50, 24.00	110	4.14 ± 2.34	1.30, 18.10	91	5.97 ± 3.96	0.50, 24.00	-4.73	<0.001
TSH	202	1.98 ± 1.07	0.09, 7.00	111	1.95 ± 0.96	0.20, 6.20	91	2.01 ± 1.19	0.09, 7.00	0.19	0.848
LH	201	5.75 ± 4.39	0.05, 42.30	111	6.77 ± 4.96	0.70, 42.30	91	4.40 ± 3.05	0.05, 16.10	5.13	<0.001
Prolactin	199	14.57 ± 7.79	1.00, 61.10	109	14.30 ± 5.42	2.30, 35.80	91	14.93 ± 10.17	1.00, 61.10	0.80	0.427
Testosterone	141	1.69 ± 0.73	0.40, 3.80	93	1.79 ± 0.83	0.40, 3.80	46	1.60 ± 0.60	0.40, 3.10	1.44	0.150
DHEAS	117	1.72 ± 0.85	0.30, 5.80	80	1.87 ± 1.07	0.40, 5.80	37	1.55 ± 0.48	0.30, 2.70	0.85	0.395
17-OHP	120	2.0 ± 0.68	0.40, 4.00	80	1.93 ± 0.63	0.40, 3.50	40	2.07 ± 0.73	0.50, 4.00	-0.91	0.364
LH/FSH	201	1.41 ± 0.97	0.02, 5.19	87	1.78 ± 0.95	0.22, 5.19	114	0.91 ± 0.74	0.02, 3.73	7.11	<0.001


PCOS; Polycystic ovary syndrome, M; Mean, SD; Standard deviation, Min; Minimum, Max; Maximum, FSH; Follicle-stimulating hormone, TSH; Thyroid-stimulating hormone, LH; Luteinizing hormone, DHEAS; Dehydroepiandrosterone sulfate and 17-OHP: 17-hydroxyprogesterone, ^a^ ; All values are in microgram per liter, and ^b^; Mann-Whitney U (Asymp. P). Bolded results indicate a significant difference.

## Discussion

The aim of this study was to evaluate sexual function and its correlates among infertile patients with and without PCOS. The findings suggest that infertile women with and without PCOS considerably suffered from diminished sexual function. A recent meta-analysis reported domains of lubrication, orgasm, and satisfaction to be the sources of difference between infertile and fertile women (24), while these three domains were proportionately better in the two groups evaluated in the current study

It should be noted that domain-specific FSD was determined by a relatively higher cut-off point (< 3.9), which was originally applied to infertile women (25); however, some other studies employing slightly lower cut-off points, reported a relatively higher degree of domain-specific FSD in healthy Iranian women (26). Nevertheless, similar to the aforementioned study (26), desire and arousal problems not only showed a marked prevalence but were also the most problematic sexual domains in the current study

In addition, the literature suggests that women with and without PCOS in the general population (16, 17) and infertile Iranian women with and without PCOS (27) do not differ in terms of sexual function. Although PCOS women compared with their healthy counterparts, may mainly have dissatisfaction with their sex life, rather than with sexual activity (28), the current study indicated a diminished level of sexual function in both groups, with the orgasm domain being a specific source of difference. This indicates a crucial need for further attention paid to the sexuality of infertile PCOS women.

Furthermore, while some studies could not determine the effects of hyperandrogenic manifestations on sexual function (29), others indicated that acne-related concerns could reduce sexual satisfaction in both PCOS women and their spouses (30). Also, in our study, although infertile PCOS patients featured higher levels of LH and FSH, as well as an elevated LH/FSH ratio, it was the non-PCOS group that showed a significant association between LH and pain. In addition, an earlier study found no association between LH and quality of life of PCOS patients (31). However, LH was previously shown to be connected with orgasm problems in healthy, postmenopausal women (32) and with sexual function in PCOS patients in the general population (33). LH contributes to the circulation of reproductive hormones, including androgens and estrogens (34), and it is suggested to make women apt to love and intimacy (35). In addition, studies in which significant hormonal correlations were seen with LH in PCOS patients, emphasized the multifactorial nature of human sexual function which can be influenced by a variety of psychosocial and cultural factors (33). Thus, more studies are needed to provide supports for the results observed in the current study.

The current study revealed a significant contrast between the two groups in the relationship between prolactin level and sexual function. Other studies have also reported that among various relevant hormones, there was only a negative association between prolactin level and function of orgasm in PCOS women (36). Elevated prolactin levels were found to be associated with sexual problems in the general population (37). Also, women with PCOS can have mildly elevated levels of prolactin (38). Thus, the current results in line with the previous findings (36), indicated the diminished function of orgasm to be associated with higher prolactin levels in infertile PCOS women.

Contrary to expectations, higher central obesity which is marked in PCOS women (11, 12), indicated lower sexual arousal in infertile non-PCOS women only. Additionally, although some studies reported that the age of infertile women had a negative association with sexual function (39), the current findings instead, suggest the negative impact of marital duration on sexual arousal and total FSFI in infertile PCOS women. Thus, the current results indicate that obesity and marital characteristics could also be sources of difference in sexual function between infertile PCOS women and those without PCOS. 

Ultimately, the distinctions raised in the current study suggest that infertile PCOS and non-PCOS women may need more well-tailored research on their specific biological, hormonal, and psychological dimensions. It is suggested that future studies include spouses assessments to further examine the relational nature of sexual desire and arousal in patients. More importantly, some studies have suggested implementing educational interventions to enhance the sexual function of infertile women (40). The current study, which indicates the exclusively negative effect of prolactin level, acne, and marital duration on the sexual function of infertile PCOS women, implies that interventions may need to be modified accordingly to educate patients on how to manage their specific problems. Last but not least, policymakers concerned with the family structure and sexual health of the Iranian population, should focus on and facilitate particular needs and problems of the patients in order to maintain their marriage as socially stable and psychologically fruitful as the wider population does.

This study lacked a control group of fertile women. Therefore, it failed to find any possible differences between fertile and infertile women, especially those who may seek professional help for their sexual problems. Moreover, since this study had a cross-sectional design, caution needs to be taken in making any generalizations or considering causal implications.

## Conclusion

This study demonstrated diminished sexual function in infertile Iranian women, especially in terms of the desire and arousal domains. The PCOS and non-PCOS groups were not significantly different in terms of sexual function, while orgasm dysfunction was higher in the PCOS women. In addition, acne increased sexual dysfunction in the PCOS women. The infertile non-PCOS women with higher levels of prolactin had lower dyspareunia and those with higher LH had lower total FSFI and lubrication problems, while the higher the central obesity the higher their arousal problems. However, infertile PCOS women mainly showed orgasm dysfunction as a result of lower levels of prolactin, and lower total FSFI and arousal as a result of marital duration.
